# A Case of Uncommon Tumor

**Published:** 1801-10

**Authors:** William Patterson

**Affiliations:** Londonderry


### *A Case of uncommon Tumor.*

To the EDITORS of the MEDICAL and PHYSICAL JOURNAL.

Gentlemen,

ON July 30, 1801, I was requefted by Mr. Brown, feveral years a furgical practitioner in this place, to fuperintend, along with our friend Dr. Scott, the extirpation of an uncommon tumor, pendant from the right labium pudendum of a poor girl, daughter to a cottager. The fubject's name is *Margaret Ruth*, aged twenty-two years, tall and robuft in her perfon, and frequently occupied in fome of the more laborious works of hufbandry. The tumor hangs by a ftalk about a fpan long, formed in the fuperior and greater part, by the teguments of the labium preternaturally elongated, and attached to a fhort neck, which feems to be in fubftance the fame with the tumor. This is of a globular fhape, nearly eleven inches in circumference, rather hard and unyielding to preffure, pretty fmooth on its furface, and excoriated on the inferior fide. As far as the teguments of the labium are concerned, the ftalk is endued with perfect fenfibility, but the neck and body of the tumor have little or none, all that they poffefs feeming to proceed from cutaneous connexion and confent.

No date can be afcertained for the commencement of the tumor; the only circumftance in this refpect which can be found out, is, that it was known to fome of the family above two years ago; but its increafe has been very flow, until within the preceding fix months, fince which its defcent and bulk have advanced rapidly. The patient is pofitive that it was not occafioned by any injury done to the part from which it grows, nor can fhe afcribe it to any particular caufe either external or internal. The catemenia are perfectly regular; and, except fome inconfiderable ftomach complaints, fhe otherwife enjoys a good ftate of health.

Though no pulfation could be felt in any part of the tumor or of the ftalk, yet, as the cafe was totally *new* to us, and as fome large morbid tumors have extraordinary veffels, it was thought prudent to furround the ftalk with two ligatures, one clofe to the neck, and another a little higher upon the elongation. The tying of thefe ligatures induced confiderable pain; and, indeed, were the meafures which alone gave any local uneafinefs from beginning to end of the treatment. The ligatures being fixed, the tumor was fevered under the lower one, by an incifion acrofs the neck, and only a few drops of blood cam came from the cutaneous veſſels. The ſimpleſt dreſſings, lint and a pledget with common ointment, were firſt put on; a full doſe of opium was then adminiſtered, and at the end of a few hours an emollient cataplaſm was added to the outward applications.

On diſſecting the tumor, which weighed twelve ounces, it was found to conſiſt almoſt entirely of an inorganic maſs, in ſmooth, white lamina, like layers of condenſed coagulable lymph, without a trace of any cavity or veſſel, ſave a tubular canal, reſembling a vein, which ran through it in a tranſverſe direction. The cutaneous covering was the only appurtenance which had vaſcular ſupplies that could be traced, and even theſe appeared to be ſcantily diſtributed. Excreſcences from the parts ſubſervient to generation in the female ſex, ſeem to have a proneneſs toward aſſuming an internal ſtructure ſimilar to that of the inſtance before us, at leaſt in relation to their ſmooth, laminated, buffy inſides. About twenty-eight years ago, I met with a caſe of Polypus in the Vagina, (ſee Edinburgh Med. Com. 1795, p. 298,) whoſe interior ſections exhibited very nearly the ſame whitiſh coloured and gloſſy appearance.

As to the ſequel of the preſent caſe, the portion of the ſtalk beneath the inferior ligature dropped of in a few days, but that under the ſuperior ligature was nearly a fortnight before it ſeparated. After this laſt ſeparation took place, the elongated teguments contracted a good deal; the wound began to cicatrize; little or no ſymptomatic fever occurred; and, in the ſpace of three weeks from the exciſion, the patient was able to walk home, to a diſtance of four or five miles from this town, without feeling the leaſt inconvenience. In conducting her cure, very little medicine was required; reſt with a cooling regimen, one or two mild laxatives, a few opiates, and occaſional emollient cataplaſms, were the leading meaſures employed throughout the whole treatment.

*Londonderry, Sept.* 5, 1801. WILLIAM PATTERSON.

